# Ultrasonic antifouling devices negatively impact Cuvier’s beaked whales near Guadalupe Island, México

**DOI:** 10.1038/s42003-022-03959-9

**Published:** 2022-09-22

**Authors:** Jennifer S. Trickey, Gustavo Cárdenas-Hinojosa, Lorenzo Rojas-Bracho, Gregory S. Schorr, Brenda K. Rone, Eva Hidalgo-Pla, Ally Rice, Simone Baumann-Pickering

**Affiliations:** 1 Scripps Institution of Oceanography, University of California, San Diego, La Jolla, CA USA; 2 Comisión Nacional de Áreas Naturales Protegidas, SEMARNAT, Ensenada, México; 3 Ocean Wise, Vancouver, Canada; 4 Marine Ecology and Telemetry Research, Seabeck, WA USA; 5 Sea Shepherd Conservation Society, Alexandria, VA USA

**Keywords:** Behavioural ecology, Conservation biology

## Abstract

Widespread use of unregulated acoustic technologies in maritime industries raises concerns about effects on acoustically sensitive marine fauna worldwide. Anthropogenic noise can disrupt behavior and may cause short- to long-term disturbance with possible population-level consequences, particularly for animals with a limited geographic range. Ultrasonic antifouling devices are commercially available, installed globally on a variety of vessel types, and are marketed as an environmentally-friendly method for biofouling control. Here we show that they can be an acoustic disturbance to marine wildlife, as seasonal operation of these hull-mounted systems by tourist vessels in the marine protected area of Guadalupe Island, México resulted in the reduced presence of a potentially resident population of Cuvier’s beaked whales (*Ziphius cavirostris*). Human activities are rapidly altering soundscapes on local and global scales, and these findings highlight the need to identify key noise sources and assess their impacts on marine life to effectively manage oceanic ecosystems.

## Abstract (web summary)

Underwater noise made by ultrasonic antifouling devices near Guadalupe Island, a marine protected area, resulted in the reduced presence of Cuvier’s beaked whales.

## Introduction

Sound is arguably the most important sensory modality for many marine animals, and rising levels of noise pollution pose a risk to marine species that rely on sound for communication, navigation, foraging, and predator avoidance^1–3^. Anthropogenic noise originates from sources such as shipping, naval operations, offshore construction, seismic exploration, oceanographic research, and fishing activities^4^, and noise impacts on marine mammals range from physiological (e.g., shifts in hearing sensitivity, elevated stress hormone levels) to behavioral (e.g., disruptions to breeding, foraging, or vocalizing)^5–7^. The adverse behavioral effects of underwater noise on other marine taxa such as fishes and invertebrates are less understood, and very few studies have been conducted on diving seabirds or marine reptiles, but many of these species also appear susceptible to noise exposure^8–11^. Underwater noise pollution is broadly recognized as a threat to marine life, and various international efforts seek to comprehensively address this global issue and provide management guidance for noise monitoring and mitigation^2^.

Research on the impacts of anthropogenic noise on beaked whales began due to publicized mass stranding events that were subsequently associated with military mid-frequency active sonar use^12–14^. Documented behavioral responses to naval sonar include cessation of echolocation, movement away from the sound source, and increased dive durations, which are all potential indicators of foraging disruption^15–17^. More ubiquitous anthropogenic sounds, such as vessel noise and shipboard echosounders (e.g., depth sounders or fish finders), have also been linked to changes in beaked whale behavior^18–20^, but impacts from many other anthropogenic signals have not yet been evaluated^21^.

Ultrasonic antifouling (UA) systems were developed as a modern solution to the issue of biofouling, which comes with high economic and environmental costs^22,23^. Biofouling mitigation has historically relied on paints to slow the accumulation of marine growth on the hulls of ships. However, environmental concerns about the leaching of toxic chemicals from these coatings resulted in the ban of certain biocides^24,25^ and led to the development of sound-based products. UA devices use transducers to produce high frequency (>20 kHz) signals that cause vibration or cavitation to disrupt and prevent the settlement of biofouling organisms^26,27^. Although these devices are promoted as an eco-friendly alternative to traditional antifouling options^28^, they emit anthropogenic noise into marine ecosystems and are not regulated.

**Fig. 1:**
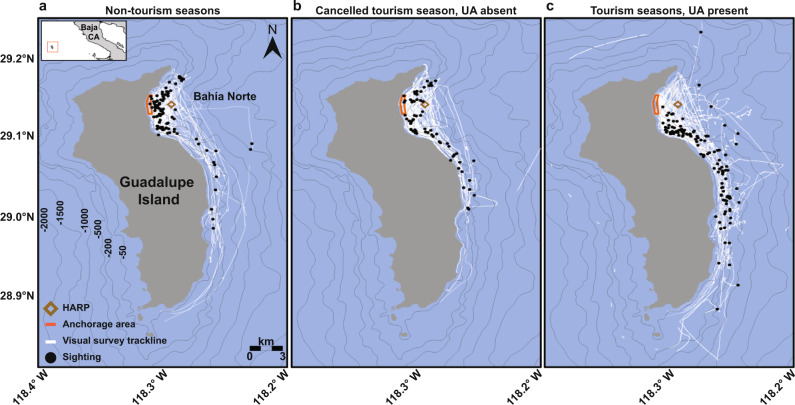
Beaked whale sighting distributions. Maps of Guadalupe Island in the Mexican waters of the Pacific Ocean (inset), illustrating the sighting locations of Cuvier’s beaked whales (black circles) and visual search effort tracklines (white lines) from cetacean surveys conducted during **a** non-tourism seasons (spring 2017 and 2019) when cage diving boats were absent, **b** a canceled tourism season (summer/fall 2020) when cage diving boats were absent due to COVID-19 restrictions, and **c** tourism seasons (summer/fall 2018 and 2019) when cage diving boats operating ultrasonic antifouling (UA) systems were present. The location of the seafloor-mounted acoustic recorder (HARP) within Bahía Norte is denoted by a brown diamond in each plot, and the anchorage area used by tourist vessels for cage diving activities is outlined in orange. Depth contours are indicated by gray lines.

**Fig. 2:**
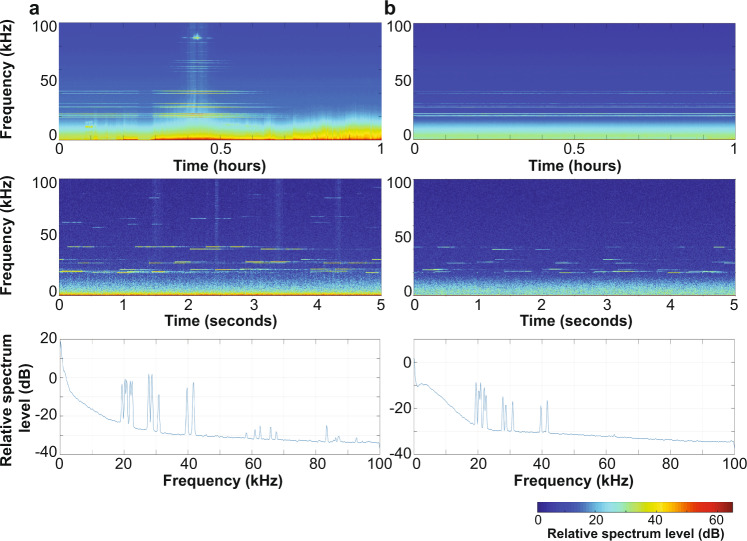
Ultrasonic antifouling signal. Long-term spectral average (top), spectrogram (middle), and spectrum (bottom) recorded on the seafloor-mounted acoustic recorder within Bahía Norte of tourist boats equipped with UA systems **a** transiting over the recorder and **b** anchored near the coastline ~2 km away. A shipboard echosounder operating at 88 kHz by the transiting boat is also present in **a**.

In this study, long-term acoustic and visual monitoring near Guadalupe Island, México revealed year-round presence of Cuvier’s beaked whales (*Ziphius cavirostris*) and suggests that this potentially resident population utilizes Bahía Norte, the large bay along its northeast coast (Fig. 1), as a foraging and breeding ground^29^. Although Guadalupe Island is a protected Biosphere Reserve with restricted access, Bahía Norte is a seasonal travel destination for great white shark (*Carcharodon carcharias*) cage diving operations throughout the summer and fall months. Several of the 11 boats with permits to conduct cage diving in 2018 and 2019 used navigational sonar systems (echosounders) while operating within Bahía Norte, and three of the boats were additionally equipped with hull-mounted UA devices (Fig. 2 and Supplementary Fig. 1). Other vessel activity during our study period included long-range recreational sportfishing boats that operated near the southern end of Guadalupe Island, scientific research vessels conducting wildlife surveys, and pangas (~7 m skiffs) used by local fishermen for artisanal lobster and abalone fisheries (Supplementary Table 1). Due to the coronavirus disease 2019 (COVID-19) pandemic, the Biosphere Reserve suspended all tourism activities at the island in 2020.

We examined long-term passive acoustic recordings collected in Bahía Norte (Supplementary Table 2) to document the acoustic presence of Cuvier’s beaked whale echolocation clicks, hull-mounted UA devices, shipboard echosounders, and motorized vessel noise from November 19, 2018 to October 3, 2020, spanning a typical cage diving tourism season at Guadalupe Island (2019) and a canceled tourism season when cage diving boats were absent (2020). Our research demonstrates that UA technology elevates underwater noise levels in frequencies used by marine mammals and resulted in a change in habitat use by Cuvier’s beaked whales at our study site.

## Results and discussion

### Patterns in beaked whale acoustic presence and distribution

**Fig. 3:**
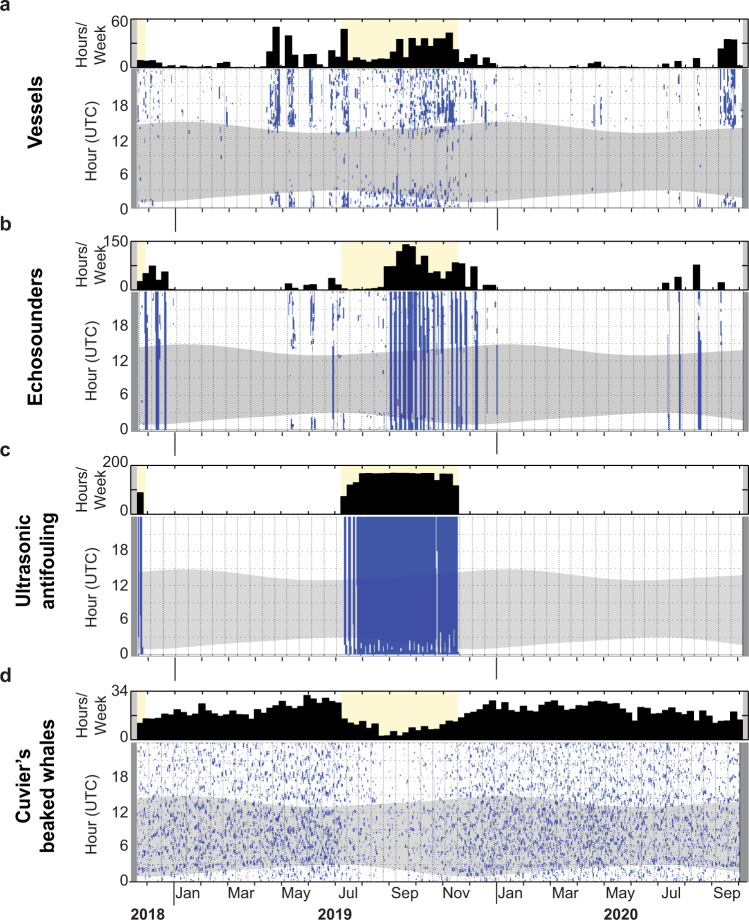
Acoustic detections. Weekly (black bars) and daily (blue dots) acoustic presence of **a** motorized vessel noise, **b** shipboard echosounders, **c** ultrasonic antifouling (UA) systems, and **d** Cuvier’s beaked whale echolocation clicks at the recording site in Bahía Norte from November 19, 2018 to October 3, 2020. Weekly plots show the number of hours of acoustic presence each week. Daily plots show acoustic presence in 1-min bins and the gray hourglass shading denotes nighttime. Gray vertical shading at the start and end of all plots denotes absence of recording effort. Yellow shading across all weekly plots in November 2018 and from July to November 2019 indicates the weeks with UA signal occurrence. A brief ~27 h effort gap in October 2019 corresponds to the time period used to refurbish the seafloor-mounted acoustic recorder.

Cuvier’s beaked whales were acoustically detected on all but two days of the two-year acoustic monitoring period at Guadalupe Island, signifying year-round presence of this species (Fig. 3d). However, beaked whale acoustic encounters sharply declined from July to November 2019, coinciding with the near-constant use of hull-mounted UA devices by some tourist vessels in Bahía Norte during the 2019 shark cage diving season (yellow shading in Fig. 3). A similar decrease did not occur in 2020, indicating that the pattern observed in 2019 was not driven by natural seasonality in beaked whale presence at this location.

Visual surveys for cetaceans conducted between 2017 and 2020 (Fig. 1 and Supplementary Table 3) also revealed a shift in beaked whale distribution out of the northern half of Bahía Norte where the tourist boats operating UA devices anchored. Cuvier’s beaked whale sightings were primarily concentrated along the northeast coast of Guadalupe Island and indicated that this species is present year-round. In the absence of tourist boats, beaked whales were sighted all throughout Bahía Norte, including relatively close to the coastline. In contrast, when tourist boats equipped with UA systems were present, beaked whales were sighted further offshore and further south along the island’s east coast. Sightings from the fall 2020 surveys, when tourism activities were canceled, demonstrated that these differences in habitat use were not due to a natural seasonal pattern (i.e., spring vs. fall), but instead appeared to be associated with UA signal occurrence. Therefore, the diminished acoustic presence of Cuvier’s beaked whales during the 2019 cage diving season did not merely reflect a change in vocal behavior, but instead suggests that exposure to the UA sound caused these potentially island-associated whales to lessen the amount of time spent in this part of their habitat.

### Characterization of the ultrasonic antifouling signal

The UA signal was composed of a suite of pulses in a range of targeted frequencies starting at ~19 kHz (Fig. 2). The signal ranged up to ~42 kHz when vessels were anchored along the Bahía Norte coastline, approximately 2–3 km away from the seafloor-mounted recorder, and up to ~88 kHz when vessels were transiting past the recorder (Fig. 2). Opportunistic recordings collected <300 m away from a tourist vessel equipped with a UA system further revealed pulses up to ~166 kHz (Supplementary Fig. 1) that were above the bandwidth of our long-term, seafloor-mounted recorder. In 2018, the UA signal was detected in November, which was the start of our acoustic recording effort and coincident with the end of the 2018 cage diving season (Fig. 3c and Supplementary Table 1). In July 2019, there was a brief ramp-up period due to the intermittent presence of only one tourist vessel at the start of the 2019 cage diving season, but by the end of that month all three of the boats equipped with UA systems were operating at the island and the UA signal was detected nearly continuously until mid-November. The only breaks in this signal occurred approximately once every five days from 00:00 to 03:00 UTC, which signified the time between the departure of one boat and the arrival of the next (Fig. 3c daily acoustic presence plot). In 2020, the UA signal was not detected at all because tourism activities were not permitted in the waters of the Guadalupe Island Biosphere Reserve.

Some UA system manufacturers erroneously claim that the frequencies emitted by the transducers cannot be heard by marine mammals, or that the ultrasonic properties of the sound cause it to remain close to the hull and not stray into open water. Indeed, the UA signal’s spectral content directly overlaps with the communication space of Cuvier’s beaked whales (Supplementary Fig. 2) and many other marine mammals^30^. Furthermore, although source levels of the UA signal were not measured, the elevated received levels at the acoustic recorder indicate that it was a sound source with a transmission range of more than 2 km, given that it was consistently detected on a seafloor-mounted recorder at 1100 m depth and at a horizontal distance >2 km away from the anchored boats. Most of the literature on UA technology focuses on its performance as an antifouling control^26,27,31^. The only peer-reviewed work to examine the impacts of UA devices on the health of a non-target marine organism found that long-term exposure to UA devices resulted in alterations to the microbiome of farmed fish^32^. Underwater noise monitoring conducted in Vancouver’s inner harbor documented elevated noise levels due to a UA system installed on a berthed cruise ship^33^, but this study did not examine any corresponding impacts on marine life.

### Other vessel-based sound sources

Noise from vessel engines occurred year-round in Bahía Norte but was most prevalent throughout the 2019 shark cage diving season and during cetacean surveys in spring 2019 and fall 2020 (Fig. 3a). Vessel detections were comprised of broadband ship noise emitted by tourist boats and research vessels, as well as noise produced by pangas, which had a different acoustic signature compared to the larger boats (Supplementary Fig. 3). Regardless of vessel type, most of the energy produced by the detected vessels was concentrated below 300 Hz. Some of the ship detections, particularly those of shorter duration that occurred during the winters in the absence of tourism and research activities, can likely be attributed to transiting sources that were not operating at the island, and may have even originated from distant commercial ships, as low-frequency noise emitted from large vessels can travel great distances underwater^4^. For most of the time that the tourist boats were present in Bahía Norte to conduct cage diving operations, they were anchored with their engines turned off. Unlike the UA devices and echosounders, which were often detected for several hours or days at a time, the vessel detections were comparatively transient (Fig. 3 daily acoustic presence plots).

Shipboard echosounders were detected at several different frequencies, although most transmitted at 28 or 50 kHz (Supplementary Fig. 4). Echosounder presence mainly corresponded with the 2018 and 2019 shark cage diving seasons (Fig. 3b), while detections in May 2019 were from a research vessel conducting a cetacean survey (Supplementary Table 3). All echosounder detections in 2020 were likely produced by sportfishing boats, which are typically only authorized to fish near the southern end of Guadalupe Island and appeared to be operating in violation of the Biosphere Reserve’s 2020 tourism closure. Echosounders have a relatively small detection range due to their high frequency content, and the prolonged encounter durations in 2020 (Fig. 3b daily acoustic presence plot) indicate that these vessels were remaining within Bahía Norte for extended periods of time rather than just transiting by.

### Ultrasonic antifouling affects beaked whale behavior

Shipboard echosounders have been shown to induce an acoustic response in beaked whales^20^, but the abrupt decline in beaked whale acoustic detections starting in mid-July 2019 strongly correlated with the onset of the UA signal specifically (yellow shading in Fig. 3), while echosounder detections only became prevalent two months later, in mid-September 2019. Vessel noise radiated from boat engines, propellers, and propulsion systems has also been shown to disrupt beaked whale foraging behavior^18,19^, but motorized vessel detections did not correlate well with the considerable reduction in beaked whale acoustic presence and had already peaked in April 2019 without causing a noticeable drop in beaked whale encounters (Fig. 3).

**Fig. 4:**
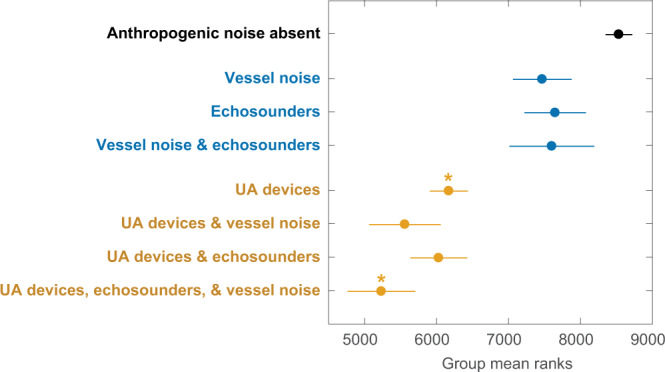
Differences in hourly beaked whale acoustic detections. Following a Kruskal–Wallis test, pairwise comparisons were computed to assess whether Cuvier’s beaked whale acoustic presence on an hourly scale differed depending on the presence/absence of the various vessel-based anthropogenic sounds. The circles and lines represent the group mean estimates and comparison intervals, respectively. Groups sharing the same color are not statistically different from one another, although asterisks denote one significant pairwise comparison (*p < 0.05). See Supplementary Table 4 for all *p* values and sample sizes.

Statistical analysis revealed significant differences in hourly beaked whale acoustic activity (Kruskal–Wallis, *χ*^2^ = 858.66, df = 7, *p* = 4.05e–181), with the highest numbers of detections occurring during quiet time periods, indicating that Cuvier’s beaked whales at Guadalupe Island had an acoustic response to all the various vessel-based anthropogenic sound sources that they encountered. However, post hoc pairwise comparisons showed that although exposure to vessel noise and/or echosounders resulted in lower numbers of detections compared to time periods lacking any anthropogenic signals, the UA devices had the strongest negative effect on beaked whale acoustic presence (Fig. 4 and Supplementary Table 4). These findings provide further evidence for the detrimental impacts of both echosounders and vessel noise on beaked whale behavior, and confirm that UA devices provoked the strongest avoidance reaction in Cuvier’s beaked whales and thus are a previously unrecognized acoustic threat to marine wildlife.

### Noise levels

**Fig. 5:**
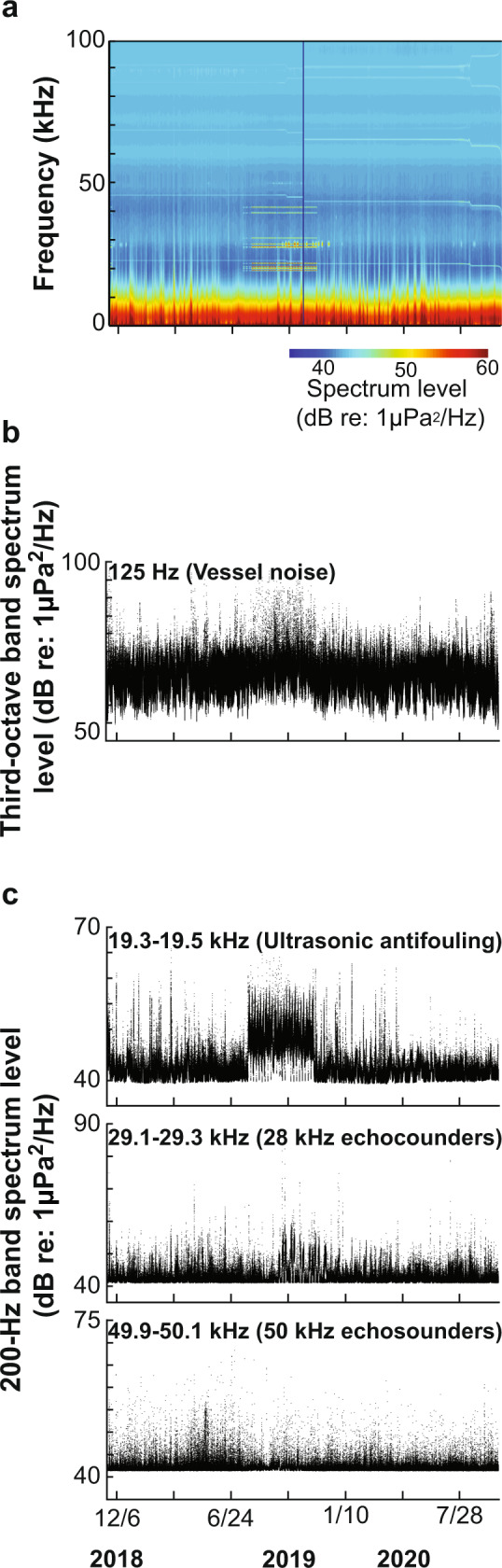
Noise levels. **a** Long-term spectrogram of acoustic data recorded in Bahía Norte from November 19, 2018 to October 3, 2020, as well as sound energy in frequency bands representative of **b** low-frequency vessel noise (nominal 125 Hz third-octave band) and **c** high-frequency signals including UA devices and echosounders transmitting at 28 kHz and 50 kHz. The minimum noise floor for the **c** measurements (~40 dB re: 1 µPa^2^/Hz) was determined by electronic self-noise produced by the acoustic recording system and was not a reflection of the true minimum ambient noise levels.

Soundscape metrics were computed, and ambient noise plots were produced for the full acoustic recording period to demonstrate the differences in noise levels and the contributions of the various anthropogenic sound sources to relevant frequency bands (Fig. 5). The UA signal was an intense component of the high-frequency soundscape at this location (Fig. 5a), and for several months in 2019 its chronic presence led to a ~7 dB increase in noise levels near 20 kHz (Fig. 5c). Although 50 kHz echosounders were a relatively common sound source in Bahía Norte, they did not substantially influence noise levels and caused only slight elevations in a frequency band representative of these narrowband, high-frequency pings (Fig. 5c). The most common echosounder frequency detected was 28 kHz, and these pings appeared to cause more considerable fluctuations in sound energy, with a ~4 dB increase in the associated noise levels during the second half of the 2019 cage diving season (Fig. 5c). Minimal changes were observed in the energy concentrated in a frequency band representative of low-frequency vessel noise (Fig. 5b), indicating that vessel engine noise did not discernibly contribute to noise levels in Bahía Norte.

### Noise pollution on a global scale

**Fig. 6:**
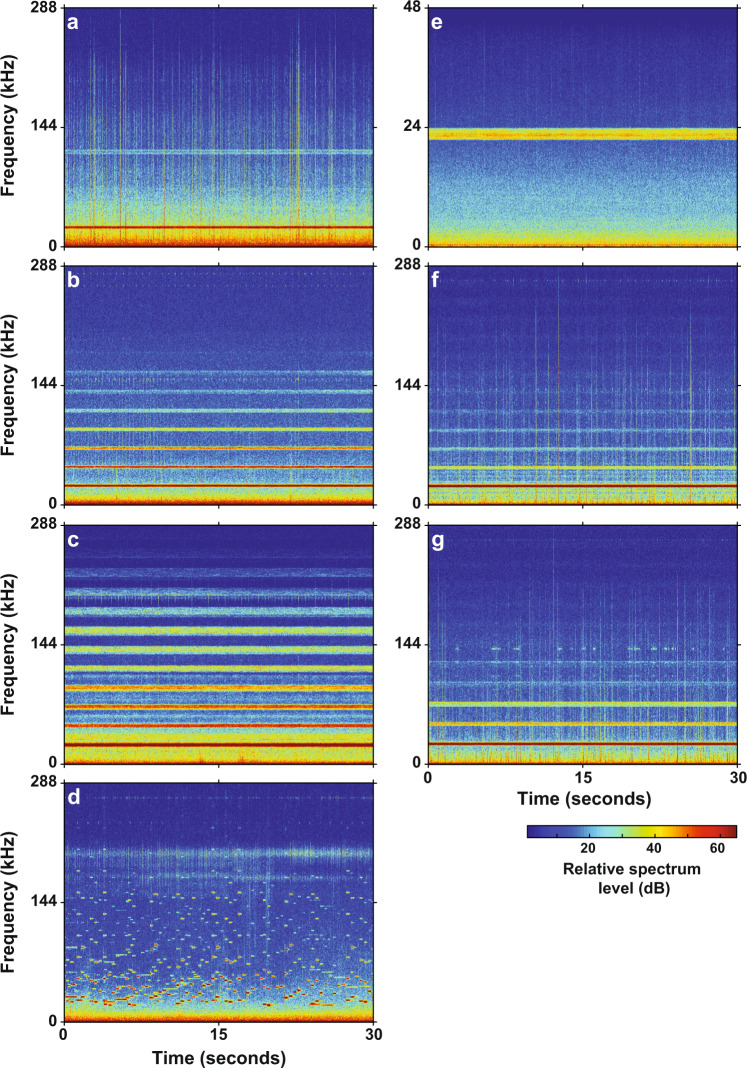
Cruise ships. Spectrograms of ultrasonic antifouling signals recorded from seven individual cruise ships from five different cruise lines, including the vessels **a** *Koningsdam*, **b** *Carnival Miracle*, **c** *Oceania Regatta*, **d** *Navigator of the Seas*, **e** *Royal Princess*, **f** *Grand Princess*, and **g** *Majestic Princess*. The recorder used for the *Royal Princess* had a 96 kHz sampling rate, and the recorders used for all other vessels had a 576 kHz sampling rate.

UA devices have become increasingly pervasive in numerous marine industries, are installed globally on a wide variety of commercial, military, and passenger vessels, and can also be used on stationary structures such as offshore wind farms, oil rigs, and aquaculture pens. Based on opportunistic acoustic recordings collected in ports near berthed cruise ships in San Diego, California, USA as well as in México in both Ensenada and La Paz (Fig. 6 and Supplementary Table 5), they appear to be especially prevalent among cruise lines, which raises concerns about the risks posed to the wildlife encountered by cruise ships and other tourist vessels utilizing this sound-based technology as they visit some of the more pristine marine areas worldwide^34^. Although the frequency content of ultrasonic (>20 kHz) antifouling devices may only be audible to animals such as odontocetes, an acoustic antifouling system that produces infrasonic (17–27 Hz) signals is also commercially available, which has implications for a diverse assortment of marine species with hearing ranges in the lower frequencies, including mysticetes, and may also be sensed by many fishes and invertebrates.

In the effort to develop an alternative solution for biofouling control, acoustic antifouling companies have traded one problem for another by substituting a noise pollutant for a chemical one. Despite manufacturer claims that this is a green technology that causes no harm to marine mammals, it produces sound at frequencies similar to those used by odontocetes for echolocation-based foraging and navigation, and our results indicate that exposure to UA systems affected the distribution of Cuvier’s beaked whales at Guadalupe Island. This sort of habitat displacement could have negative impacts on the foraging success, reproductive rates, and health of animals with high site fidelity, and could ultimately lead to a population decline^35,36^. Our findings confirm that ultrasonic antifouling can be an acoustic disturbance to marine wildlife, and serve to caution against the widespread and unrestricted use of sound-producing devices in the maritime sector without first evaluating the potential impacts of these anthropogenic noise sources on marine life. Sound is highly relevant for the health and conservation of oceanic ecosystems, and policy frameworks should consider implementing regulations on these acoustic technologies, particularly in marine sanctuaries and other biologically sensitive areas.

## Methods

### Long-term acoustic data collection

Passive acoustic monitoring was conducted from November 19, 2018 to October 3, 2020, with 683 days of recording effort overall (Supplementary Table 2), using a High-frequency Acoustic Recording Package (HARP)^37^. The HARP was deployed in Bahía Norte, Guadalupe Island, located approximately 150 miles offshore of México’s Baja California Peninsula (Fig. 1). The HARP was bottom-mounted and deployed to a depth of approximately 1100 m, with a calibrated hydrophone suspended ~30 m above the seafloor. The same hydrophone was used for both deployments to facilitate data comparison. The omnidirectional hydrophone sensor (ITC-1042, International Transducer Corporation, Santa Barbara, CA) had an approximately flat (±3 dB) hydrophone sensitivity from 10 Hz to 100 kHz of −200 dB re V/μPa. The sensor was connected to a custom-built preamplifier board and bandpass filter. The calibrated system response was corrected for during analysis. Data were sampled continuously at a 200 kHz sampling rate with 16-bit quantization, effectively monitoring a frequency range of 10 Hz–100 kHz.

### Automatic detection and manual classification of beaked whale echolocation clicks

Beaked whales can be acoustically identified by their echolocation clicks^38^. These signals are frequency-modulated (FM) upswept pulses, which appear to be species-specific and are distinguishable by their spectral and temporal features. Cuvier’s beaked whale echolocation signals are well differentiated from the acoustic signals of other beaked whale species. They are polycyclic with a characteristic FM pulse upsweep, peak frequency around 40 kHz, and uniform inter-pulse interval of about 0.4–0.5 s^39,40^. Additionally, Cuvier’s beaked whale FM pulses have characteristic spectral peaks at approximately 17 and 23 kHz.

Beaked whale FM pulses were detected in the HARP data with an automated method using the MATLAB-based (Mathworks, Natick, MA) custom software program *Triton* (https://github.com/MarineBioAcousticsRC/Triton) and other MATLAB custom routines. After all potential echolocation signals were identified with a Teager–Kaiser energy detector^41,42^, an expert system discriminated between delphinid clicks and beaked whale FM pulses. A decision about presence or absence of beaked whale signals was based on detections within a 75 s segment. Only segments with more than seven detections were used in further analysis. All echolocation signals with a peak and center frequency below 32 and 25 kHz, respectively, a duration less than 355 μs, and a sweep rate of <23 kHz/ms were deleted. If more than 13% of all initially detected echolocation signals remained after applying these criteria, the segment was classified to have beaked whale FM pulses. This threshold was chosen to obtain the best balance between missed and false detections. A third classification step, based on computer-assisted manual decisions by a trained analyst, labeled the automatically detected segments to signal type and rejected false detections^38^. The rate of missed segments was approximately 5%, varying slightly between deployments. The start and end time of each segment containing beaked whale signals was logged, and their durations were summed to calculate cumulative hourly and weekly presence.

### Manual detection of anthropogenic signals

Anthropogenic sounds, including UA devices, shipboard echosounders, and vessel engine noise, were detected by manually screening the HARP recordings with the MATLAB-based program *Triton*, using long-term spectral averages (LTSAs) displaying one hour of data at a time. The full bandwidth LTSAs (displaying data up to 100 kHz) used to detect UA devices and echosounders were created using a 5 s time average with 100 Hz frequency resolution. The mid-frequency LTSAs (displaying data up to 5 kHz) used to identify vessel noise events resulted from a decimation of the data by a factor of 20, and were created using a 5 s time average with 10 Hz frequency resolution. The start and end times of each anthropogenic signal encounter were logged, and their durations were summed to calculate cumulative hourly and weekly presence.

### Computation of soundscape metrics

Soundscape metrics of the full HARP time series were computed using the MATLAB-based *Triton* “Remora” (a type of plug-in) *Soundscape Metrics* (https://github.com/MarineBioAcousticsRC/Triton/tree/master/Remoras/Soundscape-Metrics). Periods of 15-s disk-write noise, occurring on a repeating cycle every 75 s, were omitted from analysis. The broadband (0–100 kHz) acoustic data were processed to compute power spectral density (PSD) using Welch’s method within MATLAB (FFT length = 200,000 points, Hann window length = 200,000, FFT overlap = 0%), resulting in mean-square pressure amplitude (µPa^2^) at 1-Hz, 1-s resolution. For every 1-Hz frequency band, PSD levels per 1-min were calculated as the median of mean-square pressure amplitude (µPa^2^) over no less than 30 s for that minute. PSD levels were then converted to decibels (dB re: 1 µPa^2^/Hz).

To facilitate comparison of soundscape metrics to anthropogenic signals, standard frequency bands (American National Standards Institute, ANSI 1.11-2004) were selected, and median one-third octave band sound pressure levels (TOLs) were calculated over the full time series. TOLs in units of µPa^2^ were calculated by integration of PSD estimates of the mean-square pressure (µPa^2^) with a 1-Hz, 1-s resolution over each of 39 one-third octave bands, with the nominal center frequencies ranging from 13 to 80,000 Hz (IEC 61260-1995). The resulting TOLs with 1-s resolution were then used to calculate TOLs per 1-min as a median over no less than 30 s for that minute. The TOLs per minute were converted to decibels (dB re: 1 µPa^2^). Band levels for a nominal frequency of 125 Hz were transformed to reported TOL values for comparability by adjusting for frequency band bin width (bw) by subtracting 10 × log10(bw) from band levels, resulting in dB re: 1 µPa^2^/Hz. TOL calculations centered on 125 Hz are often reported as an indicator of vessel noise, as the energy concentrated in this frequency range is representative of the continuous low-frequency sound pressure levels generated by boat engines^43^.

PSD levels per 1-min were calculated as the median of mean-square pressure amplitude (µPa^2^) over 200 Hz frequency bins and no less than 30 s for that minute. PSD levels were then converted to decibels (dB re: 1 µPa^2^/Hz). PSD levels for 19.3–19.5 kHz, 29.1–29.3 kHz, and 49.9–50.1 kHz were extracted to measure energy from specific anthropogenic sound sources, as these frequency ranges were representative of the UA signal, 28 kHz echosounders, and 50 kHz echosounders, respectively. These narrowband, 200 Hz wide frequency bins were chosen as proxies for these three anthropogenic sounds because more broadband measurements (e.g., TOLs) would also contain additional energy from other, non-target signals. Echosounders transmitting at 50 kHz produced pings with a peak frequency of 50 kHz and energy between approximately 49–51 kHz, and 28 kHz echosounder pings had a peak frequency of ~28.8 kHz and energy between approximately 27 and 31 kHz. PSD levels for 29.1–29.3 kHz were chosen as the proxy for 28 kHz echosounders to avoid any spectral overlap with an ultrasonic antifouling pulse containing energy from 28.4 to 29.0 kHz.

### Cetacean visual surveys

Twelve field trips to Guadalupe Island were undertaken between May 2017 and October 2020 at various times throughout the year (Supplementary Table 3). During these expeditions, boat-based visual surveys for cetaceans were conducted, which entailed at least two observers scanning with the naked eye for beaked whales and other cetaceans during daylight hours. Information on all cetacean sightings was logged systematically, including details such as the species, group size, and GPS location. Cetacean research activities were conducted from large vessels (17–40 m boats) and/or pangas (~7 m skiffs). Some of these field trips coincided with the 2018 and 2019 shark cage diving tourism seasons, while others occurred when tourist boats were absent from the island, such as during the spring periods of 2017 and 2019, as well as during the fall of 2020 when the tourism season was canceled due to the COVID-19 pandemic. Visual survey tracklines and sighting locations of Cuvier’s beaked whales were plotted to create maps showing search effort and sighting distributions under various conditions related to the presence of tourist vessels (Fig. 1).

### Opportunistic acoustic recordings

Opportunistic passive acoustic recordings were collected on several occasions near vessels assumed to be equipped with UA devices, using hand-deployed recorders at depths of approximately 2–7 m below the sea surface (Supplementary Table 5). In March 2021, acoustic data were collected approximately 500 m away from the *Royal Princess* cruise ship anchored in the Bay of La Paz, México. A Marantz PMD661 recorder (96 kHz sampling rate, 24-bit resolution) was used with an omnidirectional Reson TC4013.1 hydrophone (sensitivity −211 ± 3 dB re V/μPa, frequency response 1 Hz to 170 kHz, 50 kHz low-pass filter), and was programmed to record continuously at a 96 kHz sampling rate.

For all other opportunistic recordings, a SoundTrap® ST300HF recorder (Ocean Instruments, Auckland, New Zealand) was used to collect acoustic data at distances <300 m away from a variety of vessels. The ST300HF was programmed to record continuously at a 576 kHz sampling rate with a 400 Hz high-pass filter, for an effective frequency range of 400 Hz–276 kHz. With these settings, the instrument had an approximately flat frequency response (±3 dB) from 500 Hz to 150 kHz. In August 2021 at Guadalupe Island, acoustic data was collected with this recording system near the *Nautilus Belle Amie*, an anchored shark cage diving tourist vessel in Bahía Norte. This recording setup was also used to collect acoustic data on three separate occasions in October 2021 in San Diego Bay, California, USA near the berthed cruise ships *Grand Princess*, *Majestic Princess*, and *Koningsdam*, and was also used in the harbor of Ensenada, México to collect acoustic data near the berthed cruise ships *Navigator of the Seas*, *Carnival Miracle*, and *Oceania Regatta* in November 2021, December 2021, and January 2022, respectively. All opportunistic acoustic recordings were manually screened for the presence of UA signals by scanning 5 s spectrograms (1000-point FFT length, 0% overlap) of the full bandwidth data using the MATLAB-based program *Triton*.

### Statistics and reproducibility

To evaluate the impact of the vessel-based anthropogenic sound sources on Cuvier’s beaked whale acoustic presence, statistical analyses were conducted in MATLAB. Hourly sums of Cuvier’s beaked whale detections (cumulative number of minutes per hour with echolocation clicks) were calculated over the complete HARP time series, and because these data were not normally distributed a non-parametric test was applied. To examine if the number of beaked whale detections per hour differed depending on the presence/absence of the various anthropogenic signals, a Kruskal–Wallis one-way analysis of variance test was computed for hourly sums of Cuvier’s beaked whale detections under the following noise conditions: (1) UA devices, echosounders, and vessel noise absent; (2) Vessel noise present; (3) Echosounders present; (4) Vessel noise and echosounders present; (5) UA devices present; (6) UA devices and vessel noise present; (7) UA devices and echosounders present; and (8) UA devices, echosounders, and vessel noise present. Post hoc multiple comparisons were then made using the “multcompare” function in MATLAB with Bonferroni correction to determine pairwise differences between the conditions. *P* values < 0.05 were considered statistically significant.

### Reporting summary

Further information on research design is available in the Nature Research Reporting Summary linked to this article.

## Supplementary information

Supplementary Information

Reporting Summary

## Data Availability

The hourly acoustic presence data for Cuvier’s beaked whales, ultrasonic antifouling devices, shipboard echosounders, and motorized vessel noise that support the findings of this study are available on Dryad (10.6076/D1D011)^44^. Installation records from one ultrasonic antifouling system manufacturer are available for some cruise, military, and tug vessels at https://mesultra.com/customers_projects/.

## Code availability

Active versions of the code used in the acoustic data analyses are available on GitHub (https://github.com/MarineBioAcousticsRC).
