# Supplementary material for: Ultrasonic antifouling devices negatively impact Cuvier’s beaked whales near Guadalupe Island, México
Source: Commun Biol. 2022 Sep 22;5:1005. doi: 10.1038/s42003-022-03959-9 (PMC9499979; doi:10.1038/s42003-022-03959-9)
Supplement: Supplementary file 1 — Supplementary Information [file 42003_2022_3959_MOESM1_ESM.pdf]

Supplementary Information for

Ultrasonic antifouling devices negatively impact Cuvier's beaked whales near Guadalupe Island, México

Jennifer S. Trickey*, Gustavo Cárdenas-Hinojosa, Lorenzo Rojas-Bracho, Gregory S. Schorr, Brenda K. Rone, Eva Hidalgo-Pla, Ally Rice & Simone Baumann-Pickering

*Corresponding author email: jtrickey@ucsd.edu

This PDF file includes:

Supplementary Figures S1 to S4

Supplementary Tables S1 to S5

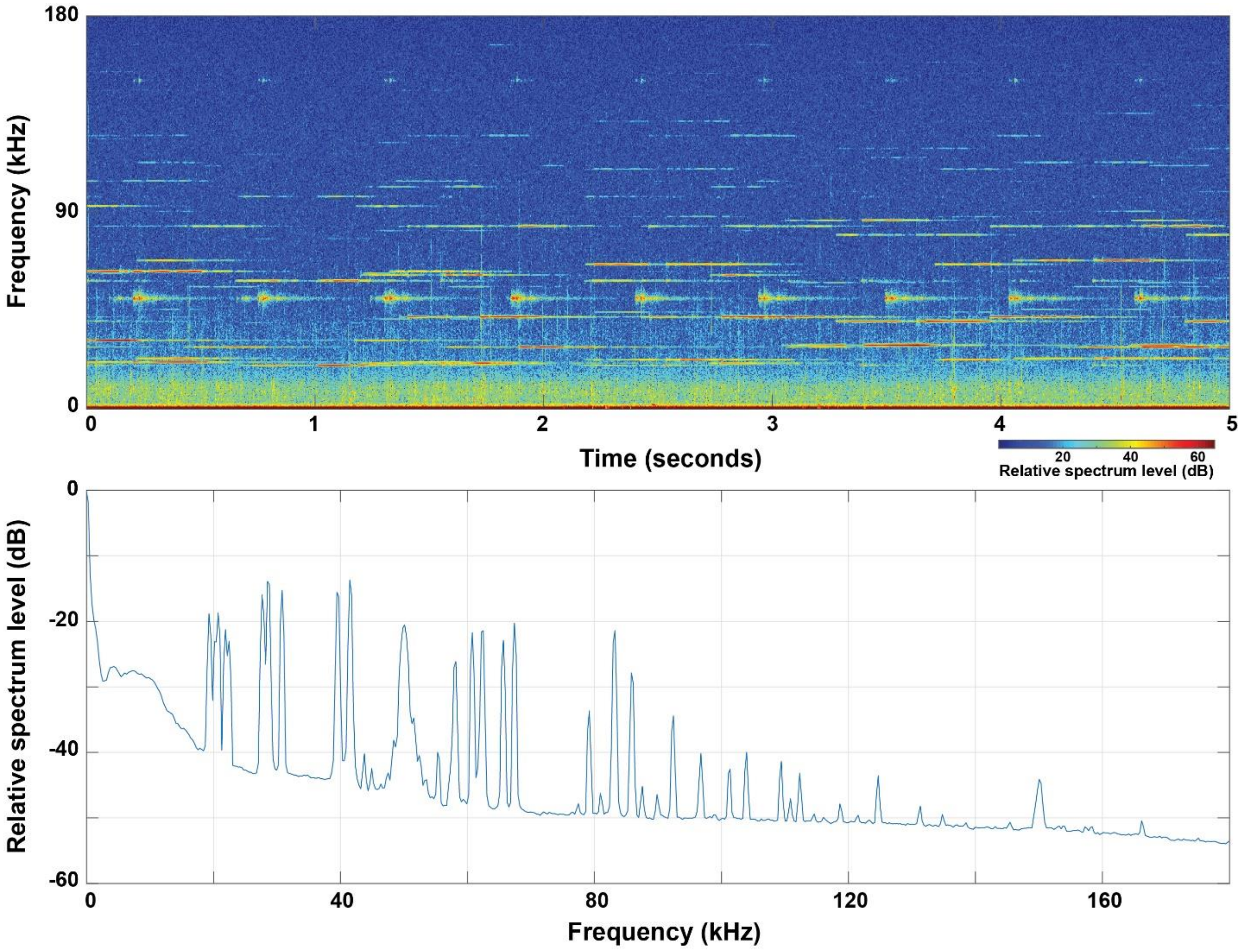

Supplementary Fig. 1

Shark cage diving tourist vessel. Spectrogram (top) and spectrum (bottom) recorded in Bahía Norte in August 2021 of an anchored tourist vessel (the *Nautilus Belle Amie*) equipped with an ultrasonic antifouling system. Shipboard echosounders operating at 50 kHz and 150 kHz are also present.

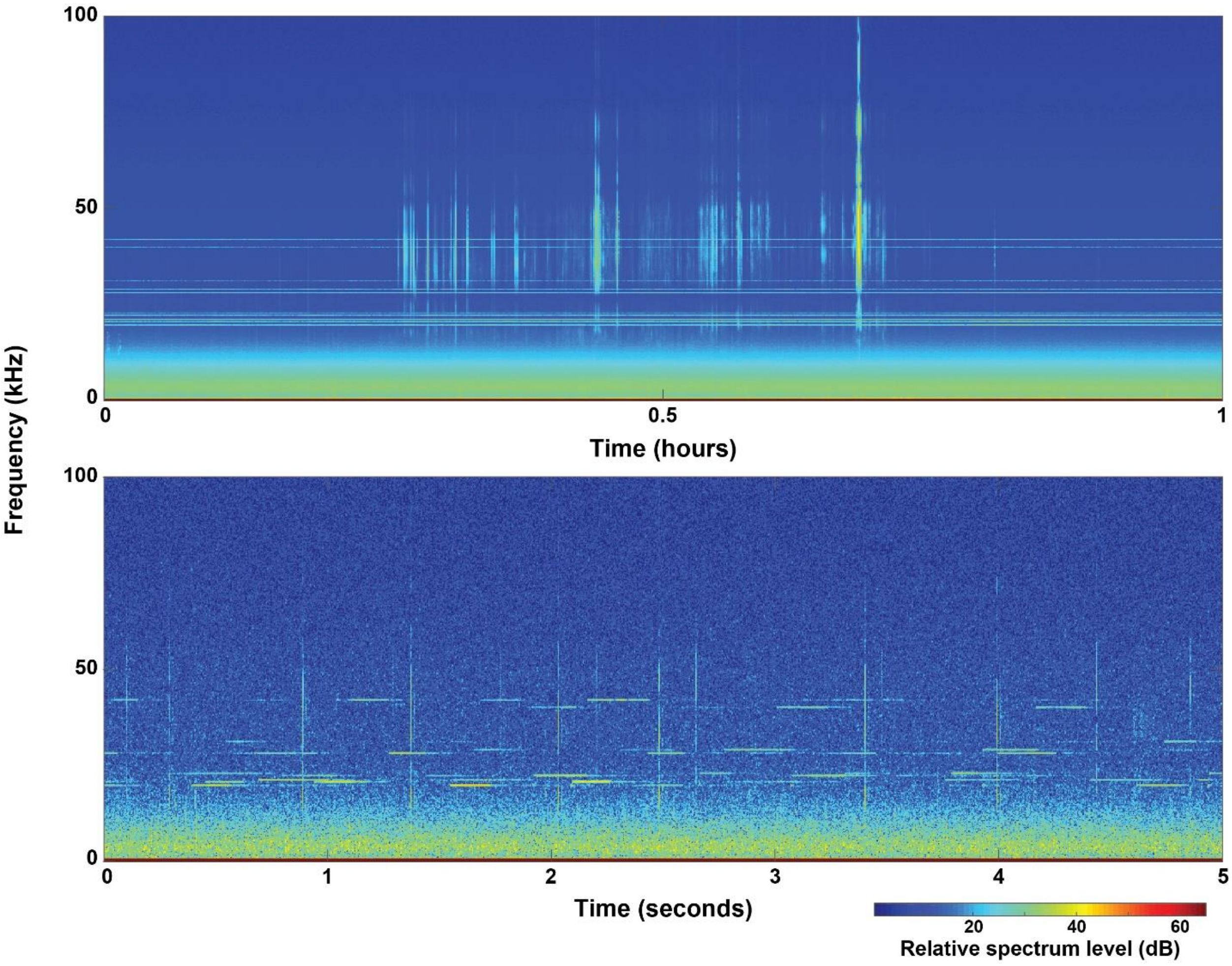

Supplementary Fig. 2

Spectral overlap. Long-term spectral average (top) and spectrogram (bottom) recorded in Bahía Norte showing that the frequency content of the ultrasonic antifouling signal (horizontal lines) directly overlaps with Cuvier's beaked whale echolocation clicks (vertical lines).

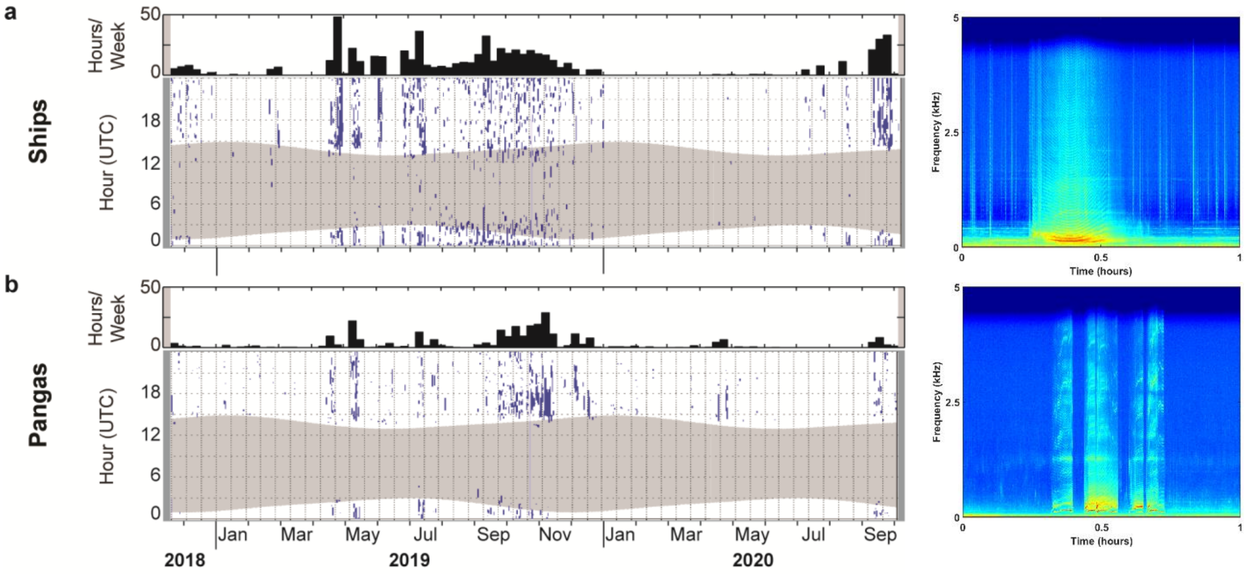

Supplementary Fig. 3

Vessel noise detections by vessel type. Weekly (black bars) and daily (blue dots) acoustic presence of motorized vessel noise emitted by **a** ships and **b** pangas at the recording site in Bahía Norte from November 2018 to October 2020. Weekly plots show the number of hours of acoustic presence each week. Daily plots show acoustic presence in one-minute bins and the gray hourglass shading denotes nighttime. Gray vertical shading at the start and end of all plots denotes absence of recording effort. Pangas (~7 m skiffs) are used by local artisanal fishermen and wildlife researchers, while most other activities are conducted from larger vessels that produce broadband ship noise (see Supplementary Table 1 for additional information on vessel activity). Example spectrograms are provided for each vessel type on the right to demonstrate the differences in their acoustic signatures.

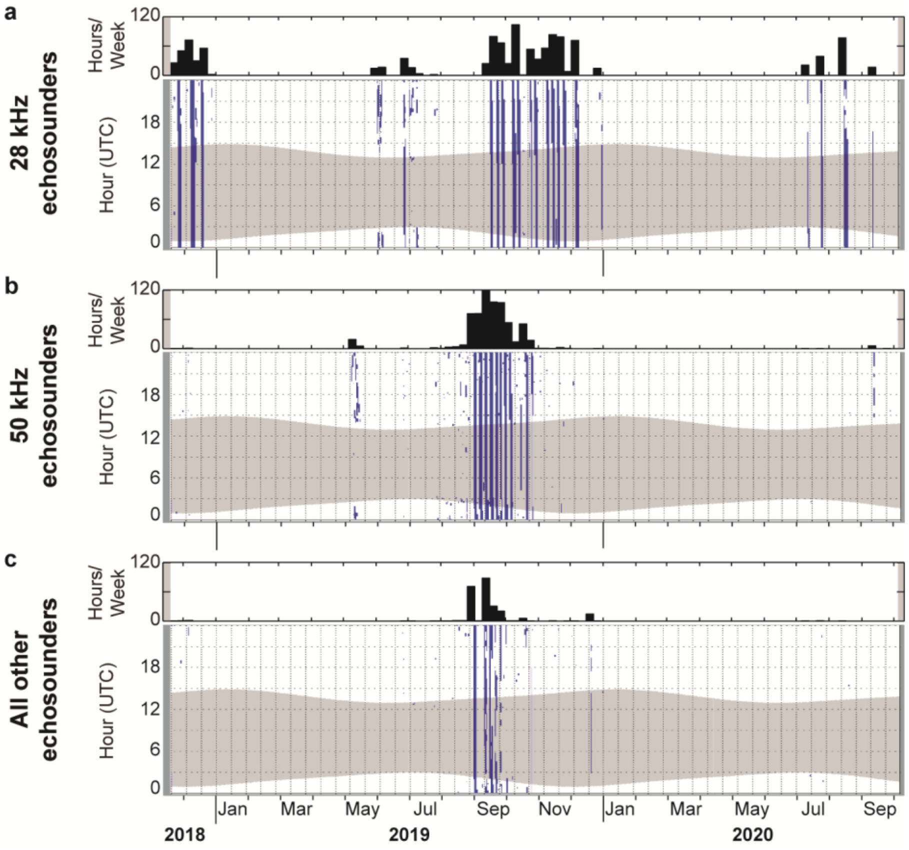

Supplementary Fig. 4

Frequency-specific echosounder detections. Weekly (black bars) and daily (blue dots) acoustic presence of shipboard echosounders operating at **a** 28 kHz, **b** 50 kHz, and **c** all other detected transmitting frequencies at the recording site in Bahía Norte from November 2018 to October 2020. Weekly plots show the number of hours of acoustic presence each week. Daily plots show acoustic presence in one-minute bins and the gray hourglass shading denotes nighttime. Gray vertical shading at the start and end of all plots denotes absence of recording effort.

Supplementary Table 1

Vessel activity at Guadalupe Island. Overview of vessel activity during the November 19, 2018 – October 3, 2020 passive acoustic monitoring period. Activities in Bahía Norte are indicated in blue. Sportfishing is indicated in orange because it is not authorized in Bahía Norte, and is only permitted near the southern end of Guadalupe Island, and thus echosounder and vessel noise detections from sportfishing boats primarily occurred when they were transiting past the recording site. See Supplementary Table 3 for more details on cetacean monitoring surveys. Pinniped monitoring was conducted from February 7–11 and August 6–14 in 2019, and seabird monitoring occurred from July 9–12, 2019. The lobster season ran from September 16 – November 30 in 2018 and 2019, and the abalone season ran from December 1 – June 30 in all years. The shark cage diving season ran from July 18 – December 6, 2018 and from July 9 – December 6, 2019; only 3 of the 11 dive boats operating during the study period were equipped with ultrasonic antifouling (UA) devices. The sportfishing season ran from July 1 – December 31, 2018 and June 30 – December 14, 2019. Artisanal fishing and research activities were conducted from pangas (~7 m skiffs), and cetacean surveys additionally used larger research vessels. Due to the COVID-19 pandemic, the shark cage diving, sportfishing, and lobster seasons were cancelled in 2020.

Year	Sector	Activity	Jan	Feb	Mar	Apr	May	Jun	Jul	Aug	Sep	Oct	Nov	Dec
2018	Research	Cetacean monitoring												
	Artisanal fishing	Lobster season												
		Abalone season												
	Tourism	Shark cage diving												
		Sportfishing												
2019	Research	Cetacean monitoring												
		Pinniped monitoring												
		Seabird monitoring												
	Artisanal fishing	Lobster season												
		Abalone season												
	Tourism	Shark cage diving												
		Sportfishing												
2020	Research	Cetacean monitoring												
	Artisanal fishing	Lobster season									cancelled			
		Abalone season												
	Tourism	Shark cage diving							cancelled					
		Sportfishing							cancelled*					

*Sportfishing boats were observed at the island throughout the summer/fall of 2020 by Biosphere Reserve personnel and cetacean researchers, and appeared to be operating illegally during the 2020 tourism closure.

Supplementary Table 2

Long-term acoustic recordings. Details of High-frequency Acoustic Recording Package (HARP) deployments in Bahía Norte, Guadalupe Island, México, including recording periods, latitudes, longitudes, depths, and recording durations. The first and last day of each deployment represent partial recording days. The HARP was recovered, refurbished with new batteries and data disks, and re-deployed over a one-day period in October 2019.

Recording period	Latitude (N)	Longitude (W)	Depth (m)	Duration (days)
11/19/2018 – 10/22/2019	29° 08.462’	118° 15.658’	1,113	337.5
10/23/2019 – 10/03/2020	29° 08.546’	118° 15.503’	1,187	345.4

Supplementary Table 3

Cetacean visual survey effort. Details of cetacean surveys conducted during the 2017-2020 study period at Guadalupe Island, México, including dates of visual surveys, the research platforms used, and whether tourist boats conducting shark cage diving operations were simultaneously present. The ultrasonic antifouling (UA) signal was only detected in passive acoustic monitoring data collected during the 2018 and 2019 tourism seasons. In addition to these research vessels (17-40 m boats), a panga (~7 m skiff) was also used for cetacean research during these survey dates.

	**Survey effort dates**	**Research vessel**	**Tourist boats present?**
Non-tourism seasons	05/15/2017 – 05/28/2017	R/V Martin Sheen	No
	04/17/2019 – 04/28/2019	R/V Martin Sheen	No
	05/07/2019 – 05/15/2019	M/V Storm	No
Tourism seasons	09/09/2018 – 09/11/2018	R/V Seas Lyfe	Yes
	10/08/2018 – 10/15/2018	M/V Storm	Yes
	10/30/2018 – 11/08/2018	R/V Martin Sheen	Yes
	11/18/2018 – 11/19/2018	R/V Martin Sheen	Yes
	07/09/2019 – 07/15/2019	M/V Storm	Yes*
	09/12/2019 – 09/17/2019	M/V Storm	Yes
	10/21/2019 – 10/29/2019	M/V White Holly	Yes
Cancelled tourism season	09/10/2020 – 09/25/2020	R/V Martin Sheen	No
	10/12/2020 – 10/27/2020	R/V Martin Sheen	No

*A tourist boat operating a UA system was only intermittently present during the July 2019 visual survey dates.

Supplementary Table 4

Pairwise comparisons of Cuvier's beaked whale hourly acoustic presence under different noise conditions. Beaked whale acoustic activity varied depending on the noise condition (Kruskal-Wallis, $\chi2 = 858.66$, df = 7, $p = 4.05e{-}181$). Post-hoc pairwise comparisons with Bonferroni adjustment are given as *p*-values, with significant ($p < 0.05$) results indicated in bold. The sample size for each noise condition is given in parentheses (there were a total of 16,390 hours, or ~683 days, of recordings overall).

Condition	2	3	4	5	6	7	8
1. Anthropogenic noise absent (11,968 hours)	**4.95e–07**	**0.000216**	**0.009913**	**6.88e–85**	**4.55e–38**	**2.16e–40**	**1.62e–51**
2. Vessel noise present (582 hours)	--	1	1	**5.58e–08**	**1.11e–09**	**6.91e–07**	**4.74e–14**
3. Echosounders present (529 hours)		--	1	**1.20e–09**	**3.93e–11**	**2.66e–08**	**1.04e–15**
4. Vessel noise & echosounders present (299 hours)			--	**1.00e–05**	**5.45–08**	**1.60e–05**	**3.30e–11**
5. UA devices present (1,557 hours)				--	0.399803	1	**0.002578**
6. UA devices & vessel noise present (401 hours)					--	1	1
7. UA devices & echosounders present (611 hours)						--	0.115066
8. UA devices, echosounders, & vessel noise present (443 hours)							--

Supplementary Table 5

Opportunistic acoustic recordings. Details of opportunistic recorder deployments, including recording locations and dates, type of recorder used, and the vessels monitored. The *Nautilus Belle Amie* was an anchored shark cage diving tourist boat, and all other vessels were berthed/anchored cruise ships in port.

Location	Date and local time	Recorder	Vessel (operator)
La Paz, México	03/29/2021 08:52	PMD661	*Royal Princess* (Princess Cruises)
Guadalupe Island, México	08/18/2021 07:03	ST300HF	*Belle Amie* (Nautilus Liveaboards)
San Diego, CA, USA	10/24/2021 06:30	ST300HF	*Koningsdam* (Holland America Line)
San Diego, CA, USA	10/24/2021 18:01	ST300HF	*Grand Princess* (Princess Cruises)
San Diego, CA, USA	10/28/2021 08:07	ST300HF	*Majestic Princess* (Princess Cruises)
Ensenada, México	11/28/2021 12:37	ST300HF	*Navigator of the Seas* (Royal Caribbean)
Ensenada, México	12/08/2021 11:15	ST300HF	*Carnival Miracle* (Carnival Cruise Line)
Ensenada, México	01/20/2022 15:50	ST300HF	*Oceania Regatta* (Oceania Cruises)
