# Supplementary material for: Ultrasonic antifouling devices negatively impact Cuvier’s beaked whales near Guadalupe Island, México
Source: Commun Biol. 2022 Sep 22;5:1005. doi: 10.1038/s42003-022-03959-9 (PMC9499979; doi:10.1038/s42003-022-03959-9)
Supplement: Supplementary file 2 — Reporting Summary [file 42003_2022_3959_MOESM2_ESM.pdf]

Corresponding author(s): Jennifer S. Trickey

Last updated by author(s): Aug 28, 2022

Reporting Summary

Nature Research wishes to improve the reproducibility of the work that we publish. This form provides structure for consistency and transparency in reporting. For further information on Nature Research policies, see our Editorial Policies and the Editorial Policy Checklist.

Statistics

For all statistical analyses, confirm that the following items are present in the figure legend, table legend, main text, or Methods section.

n/a	Confirmed	
☐	☒	The exact sample size (*n*) for each experimental group/condition, given as a discrete number and unit of measurement
☐	☒	A statement on whether measurements were taken from distinct samples or whether the same sample was measured repeatedly
☐	☒	The statistical test(s) used AND whether they are one- or two-sided<br>*Only common tests should be described solely by name; describe more complex techniques in the Methods section.*
☒	☐	A description of all covariates tested
☐	☒	A description of any assumptions or corrections, such as tests of normality and adjustment for multiple comparisons
☐	☒	A full description of the statistical parameters including central tendency (e.g. means) or other basic estimates (e.g. regression coefficient) AND variation (e.g. standard deviation) or associated estimates of uncertainty (e.g. confidence intervals)
☐	☒	For null hypothesis testing, the test statistic (e.g. *F*, *t*, *r*) with confidence intervals, effect sizes, degrees of freedom and *P* value noted<br>*Give P values as exact values whenever suitable.*
☒	☐	For Bayesian analysis, information on the choice of priors and Markov chain Monte Carlo settings
☒	☐	For hierarchical and complex designs, identification of the appropriate level for tests and full reporting of outcomes
☒	☐	Estimates of effect sizes (e.g. Cohen's *d*, Pearson's *r*), indicating how they were calculated

Our web collection on statistics for biologists contains articles on many of the points above.

Software and code

Policy information about availability of computer code

Data collection	Passive acoustic data were collected using a High-frequency Acoustic Recording Package (HARP), an instrument custom-built at the Marine Physical Laboratory at UCSD Scripps Institution of Oceanography.
Data analysis	Passive acoustic data were analyzed using MATLAB-based custom software programs. Active versions of the code used in the passive acoustic data analyses are available on Github (https://github.com/MarineBioAcousticsRC).

For manuscripts utilizing custom algorithms or software that are central to the research but not yet described in published literature, software must be made available to editors and reviewers. We strongly encourage code deposition in a community repository (e.g. GitHub). See the Nature Research guidelines for submitting code & software for further information.

Data

Policy information about availability of data

All manuscripts must include a data availability statement. This statement should provide the following information, where applicable:

- Accession codes, unique identifiers, or web links for publicly available datasets
- A list of figures that have associated raw data
- A description of any restrictions on data availability

The hourly acoustic presence data for Cuvier’s beaked whales, ultrasonic antifouling devices, shipboard echosounders, and motorized vessel noise that support the findings of this study are available on Dryad (https://doi.org/10.6076/D1D011). Installation records from one ultrasonic antifouling system manufacturer are available for some cruise, military, and tug vessels at https://mesultra.com/customers_projects/.

Field-specific reporting

Please select the one below that is the best fit for your research. If you are not sure, read the appropriate sections before making your selection.

☐ Life sciences ☐ Behavioural & social sciences ☒ Ecological, evolutionary & environmental sciences

For a reference copy of the document with all sections, see nature.com/documents/nr-reporting-summary-flat.pdf

Ecological, evolutionary & environmental sciences study design

All studies must disclose on these points even when the disclosure is negative.

Study description	This study was based on long-term passive acoustic monitoring conducted at Guadalupe Island, Mexico, as well as beaked whale sightings from dedicated cetacean visual surveys conducted at the study site.
Research sample	Passive acoustic data were sampled continuously at a 200 kHz sampling rate with 16-bit quantization, effectively monitoring a frequency range of 10 Hz - 100 kHz. Several boat-based visual surveys were also conducted to document the presence of cetaceans in the study area.
Sampling strategy	Passive acoustic data were examined to document the acoustic presence of Cuvier's beaked whale echolocation clicks, hull-mounted ultrasonic antifouling devices, shipboard echosounders, and motorized vessel noise. Beaked whale sighting locations logged from visual surveys were also examined to investigate the local distribution of Cuvier's beaked whales.
Data collection	Passive acoustic recordings were collected using an autonomous, seafloor-mounted recorder. Shipboard visual surveys for cetaceans were also undertaken at various times throughout the study period.
Timing and spatial scale	Passive acoustic monitoring was conducted from November 19, 2018 to October 3, 2020. A brief (~27 hour) effort gap occurred from October 22 to 23, 2019, when the acoustic recorder was recovered, refurbished with new batteries and data disks, and re-deployed. Twelve visual surveys for cetaceans were conducted between May 2017 and October 2020.
Data exclusions	No data were excluded from the analyses.
Reproducibility	N/A
Randomization	N/A
Blinding	N/A

Did the study involve field work? ☒ Yes ☐ No

Field work, collection and transport

Field conditions	Long-term, continuous passive acoustic recordings were collected over ~2 years using an autonomous, seafloor-mounted instrument. Visual survey effort for cetaceans entailed at least two observers scanning with the naked eye for beaked whales and other cetaceans during daylight hours.
Location	The study was conducted at Guadalupe Island, Mexico. The seafloor-mounted passive acoustic recorder was deployed to a depth of ~1,100 m in Bahia Norte (29° 08' N, 118° 15' W).
Access & import/export	All fieldwork was conducted under approval of the Secretaria de Media Ambiente y Recursos Naturales (SEMARNAT permits SGPA/DGVS/027 40/17, SG PA/DGVS/000451/18, SG P A/DGVS/008427 /18, SG PA/DGVS/0037 4/2 0, SG PA/DGVS/000595/21, SG PA/DGVS/0065 7 /21).
Disturbance	Passive acoustic monitoring is a non-invasive technique to study wildlife, and does not disturb animal behavior. Boat-based visual surveys for cetaceans were authorized by SEMARNAT wildlife permits, the Guadalupe Island Biosphere Reserve, and Mexico's National Commission of Natural Protected Areas (CONANP).

Reporting for specific materials, systems and methods

We require information from authors about some types of materials, experimental systems and methods used in many studies. Here, indicate whether each material, system or method listed is relevant to your study. If you are not sure if a list item applies to your research, read the appropriate section before selecting a response.

Materials & experimental systems

n/a	Involved in the study
☒	☐ Antibodies
☒	☐ Eukaryotic cell lines
☒	☐ Palaeontology and archaeology
☐	☒ Animals and other organisms
☒	☐ Human research participants
☒	☐ Clinical data
☒	☐ Dual use research of concern

Methods

n/a	Involved in the study
☒	☐ ChIP-seq
☒	☐ Flow cytometry
☒	☐ MRI-based neuroimaging

Animals and other organisms

Policy information about studies involving animals; ARRIVE guidelines recommended for reporting animal research

Laboratory animals	This study did not involve laboratory animals.
Wild animals	This study focused on acoustic and visual observations of Cuvier's beaked whales. No animals were captured.
Field-collected samples	This study did not involve samples collected from the field.
Ethics oversight	All fieldwork was conducted under approval of the Secretaria de Media Ambiente y Recursos Naturales (SEMARNAT).

Note that full information on the approval of the study protocol must also be provided in the manuscript.
